# Supplementary material for: Nuclear-Magnetic-Resonance-Spectroscopy-Derived Serum Biomarkers of Metabolic Vulnerability Are Associated with Disability and Neurodegeneration in Multiple Sclerosis
Source: Nutrients. 2024 Aug 27;16(17):2866. doi: 10.3390/nu16172866 (PMC11396879; doi:10.3390/nu16172866)
Supplement: Supplementary file 1 [file nutrients-16-02866-s001.zip › nutrients-3138347-supplementary.pdf]

SUPPLEMENTARY FILE

NMR Spectroscopy-Derived Serum Biomarkers of Metabolic Vulnerability are Associated with Disability and Neurodegeneration in Multiple Sclerosis

Taylor R. Wicks [1], Irina Shalaurova [2], Richard W. Browne [3], Anna Wolska [4], Bianca Weinstock-Guttman [5], Robert Zivadinov [5], Alan T. Remaley [4], James D. Otvos [2,4], Murali Ramanathan [1,5,*]

[1] Department of Pharmaceutical Sciences, State University of New York, Buffalo, NY 14214-8033, USA; trwicks@buffalo.edu
[2] LabCorp Diagnostics, Morrisville, NC 27560, USA
[3] Biotechnical and Clinical Laboratory Sciences, State University of New York, Buffalo, NY 14214-8033, USA
[4] Lipoprotein Metabolism Laboratory, National Heart, Lung and Blood Institute, National Institutes of Health, Bethesda, MD 20892, USA
[5] Department of Neurology, State University of New York, Buffalo, NY 14214-8033, USA
* Correspondence: murali@buffalo.edu; Tel.: +1-(716)-645-4846; Fax: +1-716-829-6569

Keywords: metabolic vulnerability; lipoproteins; nuclear magnetic resonance; cholesterol; branched-chain amino acids

SUPPLEMENTARY TABLES

Table S1. Results from the regression analysis of NMR-derived biomarkers with HC-RR-PMS status and presence of history of heart disease as predictors. The regression analyses adjusted for age, sex, and BMI. The generalized eta-squared (η^2) effect size measure and *p*- values are shown.

**NMR Biomarker**	**HC-RR-PMS** η^2 (*p*-value)	**Heart Disease** η^2 (*p*-value)
Valine	**.035 (< .001)**	**.027 (< .001)**
Leucine	**.030 (.002)**	**.015 (.015)**
Isoleucine	**.023 (.009)**	**.026 (.001)**
BCAA	**.037 (< .001)**	**.026 (.001)**
Alanine	**.025 (.007)**	.007 (.09)
Citrate	< .001 (.85)	.009 (.06)
Small HDL	.002 (.62)	.002 (.36)
GlycA	**.022 (.011)**	< .001 (.70)
IVX	**.016 (.04)**	.003 (.30)
MMX	**.022 (.01)**	.009 (.06)
MVX	**.038 (< .001)**	**.010 (.043)**

Abbreviations: BCAA: Branched chain amino acids; GlycA: glycated proteins; IVX: Inflammatory Vulnerability Index; MMX: Metabolic Malnutrition Index; MVX Metabolic Vulnerability Index.

SUPPLEMENTARY FIGURE 1

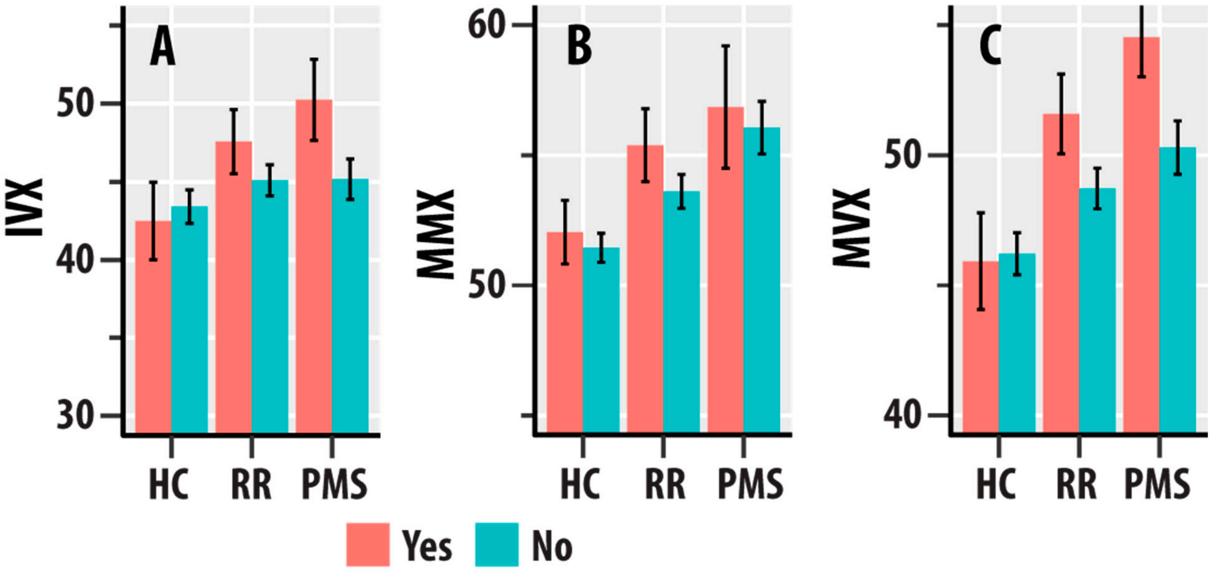

Supplementary Figure S1. Dependence of inflammation vulnerability index (IVX, Figure S1A), metabolic malnutrition index (MMX, Figure S1B), and metabolic vulnerability index (MVX, Figure S1C) on the heart problems status variable (salmon bars: Yes, teal bars: No) in healthy controls (HC), relapsing-remitting multiple sclerosis (RR), and progressive MS (PMS). The bars represent mean values, and the error bars are standard errors.
